# Improved cytotoxic effects of Salmonella-producing cytosine deaminase in tumour cells

**DOI:** 10.1111/1751-7915.12153

**Published:** 2014-09-16

**Authors:** Beatriz Mesa-Pereira, Carlos Medina, Eva María Camacho, Amando Flores, Eduardo Santero

**Affiliations:** Centro Andaluz de Biología del Desarrollo, CSIC, Junta de Andalucía, Universidad Pablo de OlavideCarretera de Utrera, Km. 1, Seville, 41013, Spain

## Abstract

In order to increase the cytotoxic activity of a *Salmonella* strain carrying a salicylate-inducible expression system that controls cytosine deaminase production, we have modified both, the vector and the producer bacterium. First, the translation rates of the expression module containing the *Escherichia coli* *codA* gene cloned under the control of the Pm promoter have been improved by using the T7 phage gene 10 ribosome binding site sequence and replacing the original GUG start codon by AUG. Second, to increase the time span in which cytosine deaminase may be produced by the bacteria in the presence of 5-fluorocytosine, a 5-fluorouracyl resistant *Salmonella* strain has been constructed by deleting its *upp* gene sequence. This new *Salmonella* strain shows increased cytosine deaminase activity and, after infecting tumour cell cultures, increased cytotoxic and bystander effects under standard induction conditions. In addition, we have generated a *purD* mutation in the producer strain to control its intracellular proliferation by the presence of adenine and avoid the intrinsic *Salmonella* cell death induction. This strategy allows the analysis and comparison of the cytotoxic effects of cytosine deaminase produced by different *Salmonella* strains in tumour cell cultures.

## Introduction

Bacteria can be easily adapted to synthesize proteins with relevant biotechnological applications. Over the past decade, many genera of bacteria have been explored as cell factories for cancer therapy due to their ability to specifically target tumours (Pawelek *et al*., 1997), reviewed in (Forbes, 2010). *Salmonella enterica* serovar Typhimurium (*S*. Typhimurium) is probably the intracellular pathogen that has been most extensively studied as an anti-tumour vector due to its intrinsic properties. These bacteria preferentially colonize and proliferate in solid tumours at ratios greater than 1000/1 compared with normal target organs, a behaviour that usually results in tumour growth inhibition (Pawelek *et al*., 1997). In addition, as a facultative anaerobe, *Salmonella* can grow under aerobic and anaerobic conditions, which allows bacteria to accumulate in large solid tumours and invade metastases (Saltzman *et al*., 1996; Yam *et al*., 2010).

Administration of attenuated *Salmonella* strains expressing different anti-tumour agents has been attempted in recent years with promising results in tumour regression (Nemunaitis *et al*., 2003; Barnett *et al*., 2005; Zhao *et al*., 2006; Royo *et al*., 2007; Jeong *et al*., 2014). One of the therapeutic genes successfully expressed in *S.* Typhimurium is the *Escherichia coli codA* gene, encoding cytosine deaminase (CD). This enzyme, present in fungi and bacteria but absent in mammalian cells (Nishiyama *et al*., 1985), catalyses the conversion of cytosine to uracil and ammonia (Koechlin *et al*., 1966). Cytosine deaminase can also deaminate the non-toxic cytosine analog, 5-fluorocytosine (5-FC) to the toxic metabolite, 5-fluorouracil (5-FU) that is widely used as a chemotherapeutic agent. This metabolite is then converted by cellular enzymes into 5-FdUMP, which inhibits DNA synthesis by blocking the activity of thymidylate synthase, 5-FUTP and 5-FdUTP, which are incorporated into RNA and DNA, respectively (Meyers *et al*., 2003), thus leading to cell death (Polak and Scholer, 1975; Damon *et al*., 2011). In addition, 5-FU can freely diffuse across the cell membrane and produce its cytotoxic effects in neighbouring cells, a phenomenon known as the bystander effect (Kuriyama *et al*., 1998). Despite several co-administration studies that have demonstrated conversion of 5-FC to 5-FU and significant tumour growth reduction in animal models (King *et al*., 2002; Nemunaitis *et al*., 2003; Royo *et al*., 2007), its application in cancer patients has been limited (Nemunaitis *et al*., 2003). Clinical data suggest that the anti-tumour activity of 5-FU is directly related to both the duration of drug exposure and its concentration in the tumour (Nemunaitis *et al*., 2003). However, in order to achieve a significant amount of active metabolites and cell killing, the required dose of the apparently harmless 5-FC may be high enough to cause adverse effects (reviewed in (Vermes *et al*., 2000)). This 5-FC toxicity may be due, in part, to the conversion of 5-FC to 5-FU by human intestinal microflora (Harris *et al*., 1986). Increasing the anti-tumour activity and minimizing the systemic toxicity would circumvent these problems, but to achieve this, it is necessary to improve the selective production of CD into the tumour. We have previously validated an *in vivo* salicylate-inducible cascade expression system that allows the controlled cytosine deaminase production. This system combines a set of salicylate-regulated elements from *Pseudomonas putida* that work in cascade, containing a regulatory module (NahR and XylS2 transcription regulators coding sequences) integrated in the chromosome of attenuated *S.* Typhimurium *aroA* (SL7207 strain) and an expression module, consisting in a *codA* gene cloned under the control of the Pm promoter either in a plasmid or integrated in the chromosome (Royo *et al*., 2007). In the presence of salicylate, XylS2 promotes transcription from Pm. In order to increase the CD production rates, in this work we have improved the CD expression module by engineering *codA* to be translated from the T7 phage gene 10 ribosome binding site and changing the original CD GUG start codon to AUG in new salicylate induced expression vectors (Medina *et al*., 2011). Since the microbial uracil phosphoribosyltransferase, encoded by *upp*, directly converts 5-FU to the metabolite 5-FUMP, from which the other toxic metabolites are produced, strains lacking this activity are more tolerant to 5-FU (Lundegaard and Jensen, 1999). To prevent killing of the producing bacteria during accumulation of toxic 5-FU, thus increasing the time span in which *Salmonella* produces CD, we have also constructed a 5-FU resistant *upp* mutant. Finally, in order to assess the effects of CD produced by improved strains and plasmids in tumour cell cycle distribution and bystander activity in long-term cell cultures, a *purD* mutation has been generated in the producer strains to avoid cell death induced by intracellular *Salmonella* proliferation (Leung and Finlay, 1991; Mesa-Pereira *et al*., 2013).

## Results and discussion

### Construction of a *Salmonella* strain with high salicylate-induced CD production rates

In order to increase the amount of CD produced keeping standard induction conditions, we improved both the producing *Salmonella* strain and the CD expression plasmid. First, we transferred the new genome-integrated regulatory module previously developed in our laboratory (Medina *et al*., 2011) to the SL7207 *Salmonella* strain, thus generating the MPO375 strain (bacterial strains and plasmid are listed in Supporting information Table S1,). This regulatory module contains a constitutively expressed *gfp* gene to track *Salmonella* during the infection process. Second, we modified this strain with the aim of avoiding host cell death induced by *Salmonella* intracellular proliferation (see below). To that end, we transduced a *ΔpurD* mutation into MPO375 to get the strain MPO376. In this way, intracellular proliferation can be controlled by the amount of adenine in the culture medium (Leung and Finlay, 1991; Mesa-Pereira *et al*., 2013). On the other hand, we constructed new plasmids with higher CD expression rates than pMPO16, the vector previously used in our laboratory to express CD (Royo *et al*., 2007). The *E. coli codA* sequence cloned in this plasmid has the original GUG start codon and its own ribosome binding site (from now on, CD_GUG_ sequence). To increase CD production, we changed the *codA* start codon to AUG and cloned the resulting sequence into the high copy number vector pMPO52 (Medina *et al*., 2011) to produce pMPO88. In this vector, *codA* expression is under the control of the Pm promoter and the T7 gene10 ribosome binding site, a strong ribosome binding site that achieves high translation levels (from now on, CD_7AUG_ sequence). We have previously reported that the salicylate-induced expression levels of vectors based in pWSK29, a low copy number vector that is stable through the whole *Salmonella* infection cycle without selection pressure, are comparable to that of their corresponding high copy number vectors (Medina *et al*., 2011). To generate versions of the CD expression modules in low copy number plasmids, the engineered *codA* genes in pMPO88 and pMPO16 were subcloned in the pWSK29 derived vector pMPO20, thus generating plasmids pMPO90 and pMPO1088 respectively.

**Fig 1:**
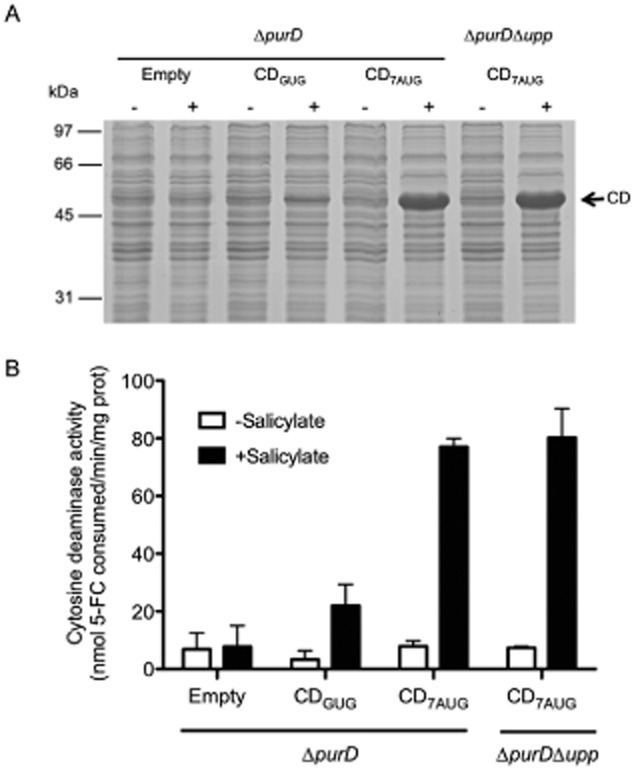
Production of CD in low copy number expression vectors.A. SDS-PAGE analysis of salicylate dependent overproduction of cytosine deaminase. Whole extracts of *Salmonella* MPO376 (Δ*purD*) bearing pMPO54 (empty vector), pMPO1088 (CD_GUG_) or pMPO90 (CD_7__AUG_) plasmids and *Salmonella* strain MPO378 (Δ*purD*Δ*upp*) bearing pMPO90 plasmid, either uninduced (-) or induced by salicylate for 4 h (+). Three μl of supernatant was loaded in each track.B. Analysis of conversion of 5-FC. Cytosine deaminase activity from cell extracts of *Salmonella* MPO376 bearing pMPO54, pMPO1088 or pMPO90 plasmids, and MPO378 bearing pMPO90 either induced by salicylate for 4 h, or not induced. Cytosine deaminase activity was assayed as previously described (Nishiyama *et al*., 1985). Each bar represents the average of three independent experiments ± SD.

To compare the amount of CD produced by the different constructs, we analysed whole-cell protein extracts from cultures of the strain MPO376 carrying the low copy number vectors pMPO1088 (CD_GUG_) or pMPO90 (CD_7AUG_) by SDS-PAGE, in the presence or absence of salicylate. As shown in Fig. 1A (lanes 4 and 6), the pMPO90 vector produces more CD than pMPO1088 after salicylate induction. Afterwards, we determined the CD activity from these cell lysates by analysing the conversion of 5-FC to the cytotoxic agent 5-FU. The assays (Fig. 1B) revealed that, upon induction, the strain harbouring the pMPO90 (CD_7AUG_) vector reached an activity about 3.5-fold higher than the same strain bearing pMPO1088 (CD_GUG_). Thus, these results demonstrate that the new CD_7AUG_ sequence produces higher amounts of CD and therefore 5-FU than CD_GUG_ using the same *Salmonella* producer strain and concentrations of salicylate and 5-FC.

### Expression of CD with a 5-FU resistant *Salmonella* strain

**Fig 2:**
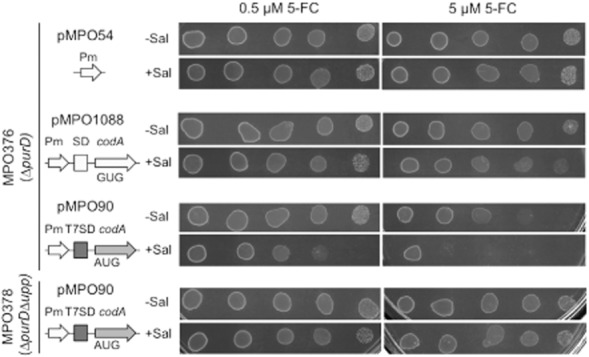
Effect of 5-FU produced on the bacterial growth. Serial dilutions (10^2^ to 10^6^) of cultures grown with or without salicylate were plated in supplemented minimal E medium in the presence of 0.5 μM or 5 μM of 5-FC and incubated for 24 h at 37°C.

As shown before, the new vector pMPO90 (CD_7AUG_) allows production of a larger amount of CD than the former construct under salicylate induction. However, since *Salmonella* is sensitive to 5-FU, the maximum rate of synthesis of this cytotoxic metabolite could also be limited by the maximum tolerated concentration and not only by the amount of CD present in the bacterium. To test this prediction, we first determined the growth of *Salmonella* carrying different plasmids on plates containing 5-FC (Fig. 2). Cultures of strain MPO376 bearing either the empty vector or one of the two CD-expressing plasmids (pMPO1088 or pMPO90) were spotted on plates in the presence or absence of salicylate and supplemented with two different concentrations, 0.5 or 5 μM, of 5-FC. Consistent with the results mentioned above, the *Salmonella* strain carrying the plasmid pMPO90 presents a more severe growth defect even at the low 5-FC concentration than the strain bearing pMPO1088 at the high 5-FC concentration, which correlates with its higher expression of CD and, consequently, higher production rate of 5-FU. This clearly showed that 5-FU production could be limited by the bacterium sensitivity to it.

This observation prompted us to obtain a *Salmonella* mutant resistant to 5-FU, strain that could produce higher amounts of this drug and for a longer time than the isogenic sensitive strain using the same 5-FC dosage. The mutant was constructed by deleting the *upp* gene sequence, whose product is involved in 5-FU sensitivity (Glaab *et al*., 2005), thus generating the strain MPO378 (Δ*purD*Δ*upp*). For the construction of *upp* mutant strain, the ‘One Step Deletion’ approach was used to replace target gene by the chloramphenicol resistance cassette (Datsenko and Wanner, 2000). We transformed this strain with the plasmid pMPO90 (CD_7AUG_) and performed the same experiments to determine the amount of CD produced and the activity of whole-cell extracts of salicylate induced cultures (Nishiyama *et al*., 1985). As shown in Fig. 1A and B, CD production and activity were independent of the *upp* mutation, since the strain behaved as its *upp*^+^ counterpart. Conversely, and as expected, the mutant was resistant to the 5-FU produced when grown on plates supplemented with 0.5 and 5 μM of 5-FC (Fig. 2) despite the high CD activity achieved. These results suggest that this strain and plasmid combination may represent an improvement in bacterial cancer therapy since it has the capacity of achieving a higher 5-FU concentration with a low 5-FC dosage, which, in turns, would reduce the deleterious effect of this compound in healthy eukaryotic cells.

### A novel strategy to analyse the cytotoxic effect of *Salmonella*-producing 5-FU in tumour cell cultures

Next, we decided to compare the consequences of 5-FU-controlled production by the different plasmids and strains obtained in this work in eukaryotic cell cultures. To determine the effects of 5-FU in eukaryotic cells, it is necessary to analyse the evolution of cell cultures for 6 days after the addition of this compound (Erbs *et al*., 2000; Bourbeau *et al*., 2004). However, once *Salmonella* has infected the eukaryotic cells, bacterial proliferation and expression of certain bacterial proteins during the first hours of infection induce host cell death within 18–24 h, hindering the study of the effect of the 5-FU produced by *Salmonella* in cell cultures (Kim *et al*., 1998; Paesold *et al*., 2002; Mesa-Pereira *et al*., 2013). To analyse the effects of the 5-FU overproduced by *Salmonella* in cell cultures, we generated a mutation in the producer strain to prevent bacterial growth and protein production inside host cell. It has been previously reported that attenuated *purD* mutants are invasion proficient but unable to proliferate once inside the eukaryotic cell. Nevertheless, the addition of adenine to culture medium can temporally suppress this deficiency (Leung and Finlay, 1991); thus, in a *purD*^-^ background, intracellular proliferation and CD overproduction can be controlled by the presence of adenine and salicylate respectively. This strategy has been recently exploited in our laboratory to study the role of SpvB *Salmonella* effector protein in the infection process (Mesa-Pereira *et al*., 2013). In the present work, we have used a similar experimental approach to study CD overproduction effects, and included a Δ*purD* mutation in all the strains used in this work. The strain MPO376 (Δ*purD*) bearing the empty vector, pMPO1088 (CD_GUG_) or pMPO90 (CD_7AUG_) and the strain MPO378 (Δ*purD*Δ*upp*) carrying pMPO90 were used to infect HeLa cells. After invasion, adenine was added to infected cell cultures. and 1 h later, once infection was established, *codA* expression was induced with salicylate. Five hours later, adenine concentration was reduced 40-fold to avoid bacterial proliferation, 50 μM of 5-FC was added and cells were incubated for 6 days in the presence of salicylate and 5-FC. As a control, uninfected cell cultures followed the same treatment but in the presence of 10 μM of 5-FU (Erbs *et al*., 2000). The effects of *codA* overexpression and 5-FU production were analysed by flow cytometry and microscopy.

**Fig 3:**
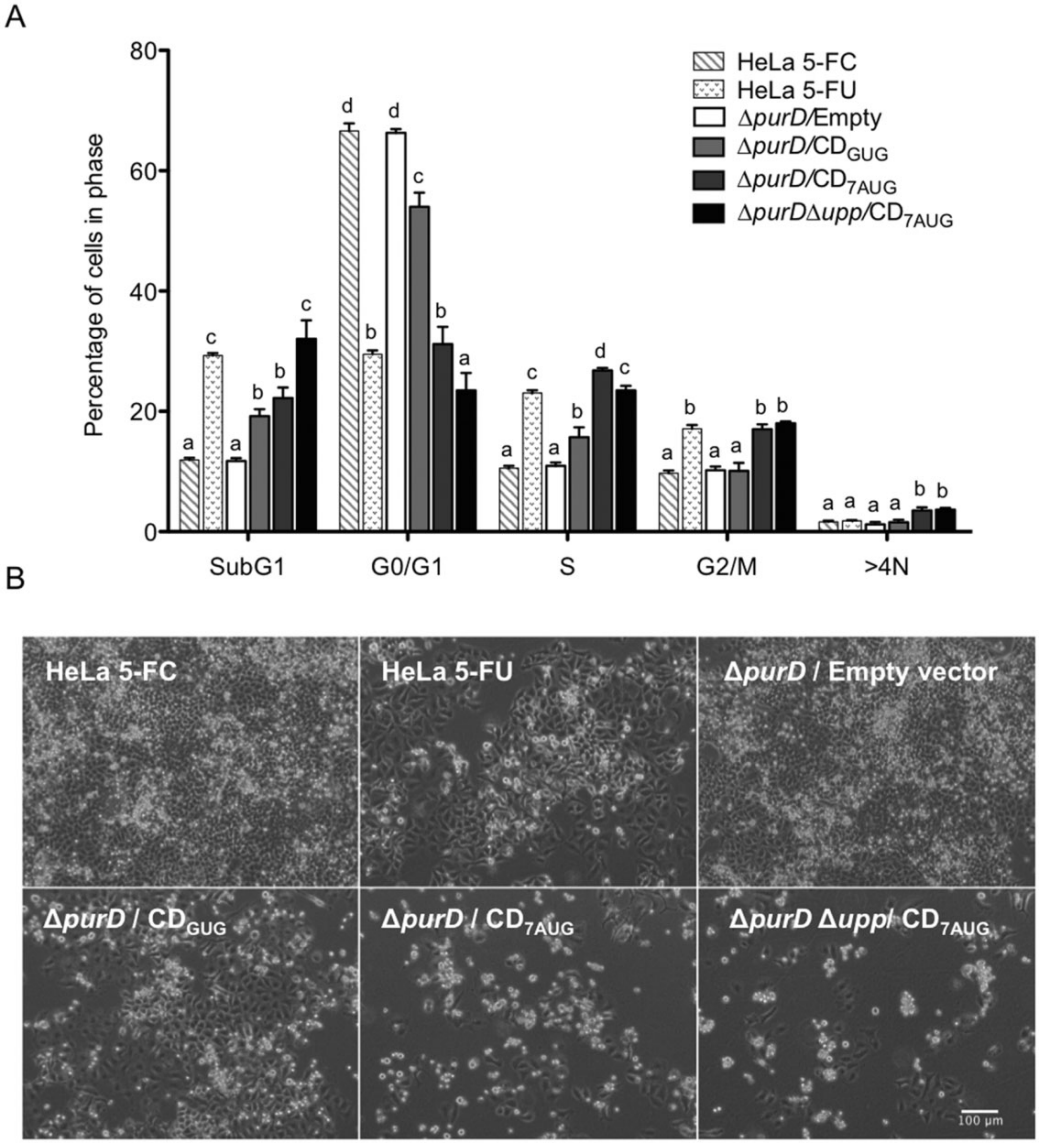
*In vitro* sensitivity to 5-FU produced by *Salmonella* on infected HeLa cells.A. Cell cycle distribution of HeLa cells infected with *Salmonella* MPO376 (Δ*purD*) bearing pMPO54 (empty vector), pMPO1088 (CD_GUG_) or pMPO90 (CD_7__AUG_) and MPO378 (Δ*purD*Δ*upp*) bearing pMPO90, at multiplicity of infection 50:1. The cells were cultured in the presence of 50 μM of 5-FC and harvested at 6 days post-induction. Ten thousand events were analysed by flow cytometry for each sample. Graphics represents the mean ± SD of three independent experiments. Non-infected HeLa cells treated with 50 μM of 5-FC or 10 μM of 5-FU were used as controls. One-way analysis of variance and Tukey HSD *post hoc* tests were applied to test for significant differences. Data from the same group marked with different alphabet are significantly different at *P* < 0.05.B. Phase contrast microscopy of infected HeLa cells as well as uninfected control and HeLa cells treated with 5-FU are shown at day 6.

Figure 3A shows the cell cycle distribution of HeLa cell cultures. As expected, most of the cells (67%) of the control HeLa cell cultures in presence of 5-FC and absence of 5-FU were in G0/G1 phase of the cell cycle. Similarly, treatment with 5-FU produced the expected effects on the cell cycle distribution (Pizzorno *et al*., 1995; Takeda *et al*., 1999; Yoshikawa *et al*., 2001; De Angelis *et al*., 2006): cells in G0/G1 phase were reduced down to 30%, while the dead cell population, represented as the percentage of cells in sub-G1 phase of the cell cycle, and cells arrested in S and G2/M phase were increased. Conversely, cell cycle of cells infected with the Δ*purD* strain bearing the empty vector were not affected even 6 days after infection, which confirms that this mutant is unable to induce cell death in the absence of adenine. Interestingly, expression of CD by this proliferation deficient strain led to a substantial alteration of the cell cycle distribution in a way similar to 5-FU (increase in dead or arrested cells and decrease in normal cells). Consistent with the above experiments, infection with the Δ*purD* strain bearing pMPO1088 (CD_GUG_) had a slight but observable effect on cell cycle distribution. However, the effect was much higher when the infecting bacteria harboured pMPO90 (CD_7AUG_). In this case, there even was an increase in > 4N cells indicative of aberrant endoreduplication. In addition, the maximum effect on cell cycle distribution was achieved when the strain bearing pMPO90 (CD_7AUG_) was the 5-FU resistant strain Δ*purD*Δ*upp* (more cells in sub-G1 than in G0/G1 phase), which was even more pronounced than that induced with 10 μM of 5-FU in the control culture. Accordingly, phase contrast microscopy showed much less proliferating HeLa cells when *Salmonella* expressed CD from pMPO90 (CD_7AUG_), an effect that was even more evident in these conditions than in 5-FU treated cultures (Fig. 3B). Additionally, to test whether the 5-FU produced from these vectors and strains has similar consequences on different tumour cell lines, we performed the same analysis to determine the effects on MCF-7 and HCT116 cell cycle distribution. As shown in Fig. S1 (Supporting information), CD controlled expression produced similar effects on MCF-7 and HCT116 cell cycle, thus showing they are not restricted to HeLa cells. Taken together, these results demonstrate that upon salicylate induction, *Salmonella* bearing the new *codA* expressing construct produces more CD and converts more 5-FU than the same strain transformed with the former construct, which subsequently correlates with a higher cytotoxicity in HeLa, MCF-7 and HCT116 cells. These high levels of 5-FU reached are also toxic to the producer strain, although using an *upp* mutant to produce CD circumvents this limitation. Finally, this experimental approach combining the salicylate induced expression system, and the *purD* mutant has proven to be effective to analyse and compare the effect of different CD producing strains on eukaryotic cell cycle. Thus, it can be a useful tool to investigate the consequences on cell physiology of any other cytotoxic protein produced by *Salmonella*, evaluate its potential as anticancer therapy agent in cell cultures and select the most appropriate combination of strains and plasmids prior to their study in animal models.

### Bystander activity of the 5-FU produced in cell cultures

Although *Salmonella* is able to invade tumour cells *in vitro*, there is some controversy regarding bacterial localization *in vivo* and some data indicate that bacteria also proliferates extracellulary in the necrotic region of solid tumour (Westphal *et al*., 2008; Crull *et al*., 2011). Therefore, the effectiveness of CD expressed by *Salmonella* in cancer therapy depends on the bystander activity of the produced drug. 5-fluorouracil has such bystander activity since it passively diffuses from cell to cell. For that reason, we compared the bystander effect on HeLa cell cultures infected with the strain MPO376 (Δ*purD*) bearing the empty vector, pMPO90 (CD_7AUG_) or pMPO1088 (CD_GUG_) and the strain MPO378 (Δ*purD*Δ*upp*) carrying pMPO90. Cytosine deaminase expression was induced with salicylate as described above but, in this case, adenine was always present in the cultures at normal concentration. Forty hours after infection, supernatants of the different cultures were transferred to uninfected HeLa cells, and cell cycle distribution was analysed by flow cytometry 6 days later. Since supernatant transfer to fresh cultures resulted in a fivefold dilution, we used uninfected cultures treated with either 10 μM or 50 μM 5-FU as control, so they were also diluted to about 2 μM and 10 μM respectively.

**Fig 4:**
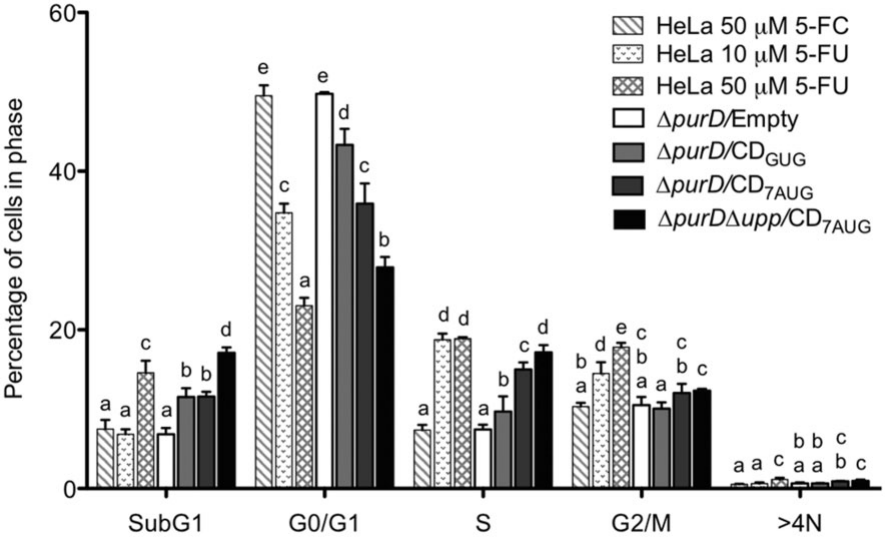
Bystander effect: Cytotoxicity of supernatants from infected HeLa cells treated with 5-FC. HeLa cells were infected at multiplicity of infection 50:1 and were grown in presence of 5-FC 250 μM in medium supplemented with adenine. Media were collected 40 h later, diluted 1:5 and added to uninfected HeLa cells. Effect of 5-FU produced was determined at day 6 by cell cycle analysis. Graphics represents mean ± SD of three independent experiments. Non-infected HeLa cells treated with 50 μM of 5-FC, 10 μM and 50 μM of 5-FU were used as controls. One-way analysis of variance and Tukey HSD *post hoc* tests were applied to test for significant differences. Data from the same group marked with different alphabet are significantly different at *P* < 0.05.

The experiments, summarized in Fig. 4, revealed a bystander effect of the three CD-expressing *Salmonella* when compared with the strain carrying the empty vector. An increase in the sub-G1 population can be observed with these three strains. Although there is little difference between the effects detected with the Δ*purD* strain carrying either pMPO90 (CD_7AUG_) or pMPO1088 (CD_GUG_), the higher effect was again achieved with the Δ*purD*Δ*upp* strain carrying pMPO90 plasmid. In fact, the consequences on cell cycle distribution caused by this combination of strain and plasmid were even greater than those generated in the control cultures.

Current gene delivery systems have low efficiency targeting tumour tissues and can transduce only a small percentage of cells within a tumour. In consequence, the clinical use of cancer gene therapy is limited. Gene therapy approaches to express CD by transformed tumour cells could surpass this limitation due to the bystander effect of the CD/5-FU system. Given that CD has a cytosolic location, a possible limitation of this approach is that CD-expressing cells are killed before cytotoxic concentrations of extracellular 5-FU are reached, limiting in this way the bystander effect and, therefore, the anti-tumour efficiency (Lawrence *et al*., 1998). Better results would probably be obtained by expressing a secreted form of CD (Rehemtulla *et al*., 2004) or, specially, using *Salmonella* as a delivery vector, because it selectively targets tumours and preferentially colonizes extracellular compartments (Pawelek *et al*., 1997; Agorio *et al*., 2007; Loessner *et al*., 2007; Leschner and Weiss, 2010; Crull *et al*., 2011). Bacterial production of CD also has limitations since *Salmonella* is also sensitive to the high concentrations of 5-FU achieved. However, the *upp*^-^ 5-FU resistance mutation in *Salmonella* would make the bacterial strain more competent in cancer therapy as it circumvents the suicide of the drug ‘factory’ and increases the bystander effect. The results of this work show that a *Salmonella* strain that combines high production levels of CD such as those achieved with pMPO90 with resistance to 5-FU due to the *upp*^-^ mutation is a better candidate to be used to intra-tumourally delivered 5-FU in cancer treatment.

## Supporting Information

**Fig. S1.** *In vitro* sensitivity to 5-FU produced by *Salmonella* on infected MCF-7 and HCT116 cells**.** A cell cycle distribution of MCF-7 (A) or HCT116 (B) cells infected with *Salmonella* MPO376 (Δ*purD*) bearing pMPO54 (empty vector), pMPO1088 (CD_GUG_) or pMPO90 (CD_7AUG_) and MPO378 (Δ*purD*Δ*upp*) bearing pMPO90, at multiplicity of infection 50:1. The cells were cultured in the presence of 50 μM of 5-FC and harvested at 6 days post-induction. 10 000 events were analysed by flow cytometry for each sample. Graphics represents the mean ± SD of three independent experiments. Non-infected cells treated with 50 μM of 5-FC or 10 μM of 5-FU were used as controls. One-way ANOVA and Tukey HSD *post hoc* tests were applied to test for significant differences. Data from the same group marked with different alphabet are significantly different at *P* < 0.05.

**Table S1.** Bacterial strains and plasmids used in or constructed for this study.
