# Supplementary figures and images for: Improved cytotoxic effects of Salmonella-producing cytosine deaminase in tumour cells
Source: Microb Biotechnol. 2014 Sep 16;8(1):169–76. doi: 10.1111/1751-7915.12153 (PMC4321383; doi:10.1111/1751-7915.12153)

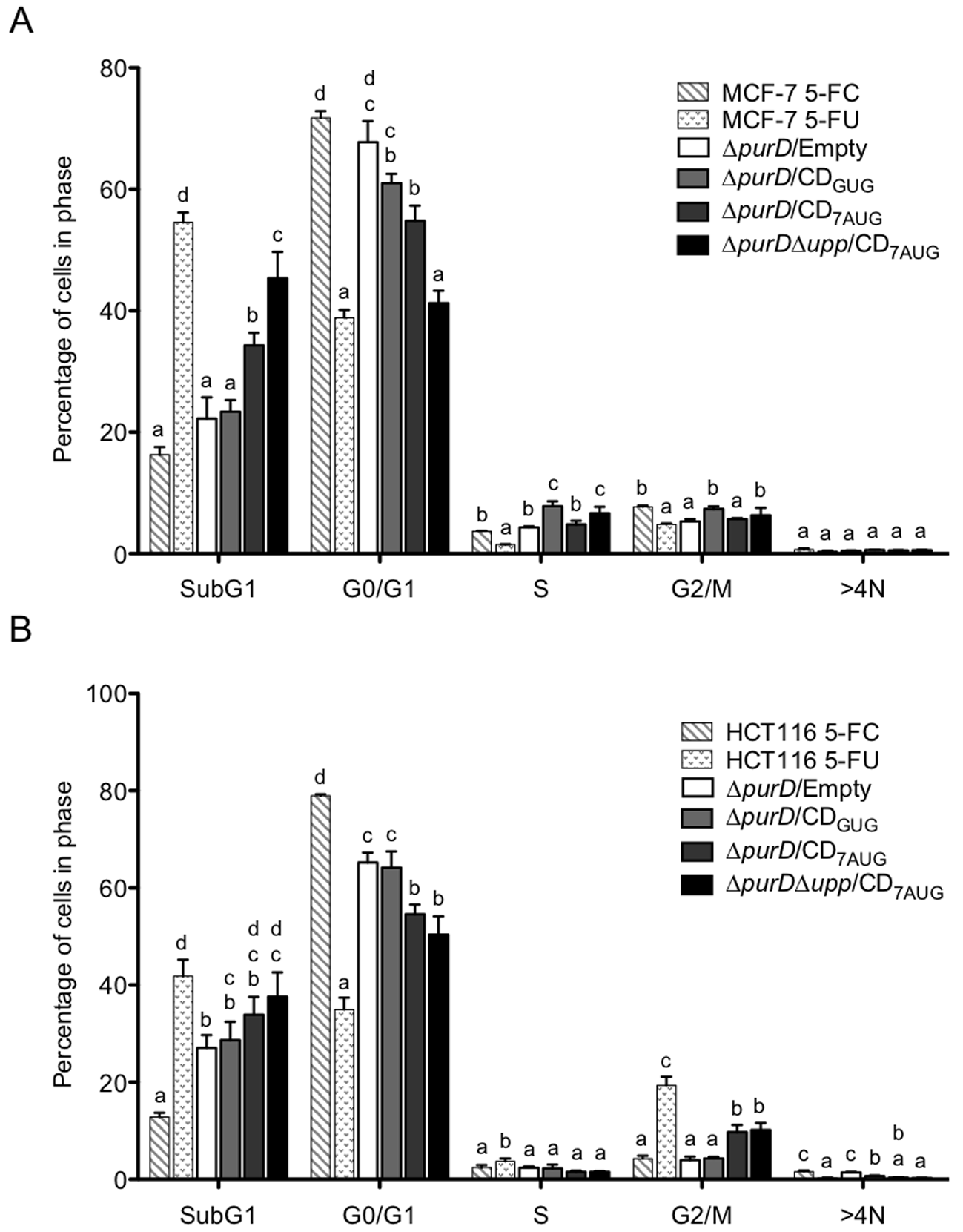

Supplement: Supplementary file 1 [file mbt20008-0169-sd1.tif]
