# Supplementary material for: Improved cytotoxic effects of Salmonella-producing cytosine deaminase in tumour cells
Source: Microb Biotechnol. 2014 Sep 16;8(1):169–76. doi: 10.1111/1751-7915.12153 (PMC4321383; doi:10.1111/1751-7915.12153)
Supplement: Supplementary file 2 [file mbt20008-0169-sd2.docx]

**Table 1. Bacterial strains and plasmids used in or constructed for this study**

| **Strain or plasmid** | **Relevant properties** | **Reference** |
| --- | --- | --- |
|  |  |  |
| ***Salmonella* strains** |  |  |
| SL7207 | hisG46 Δ407[aroA::Tn108{Tc^s^}] | ([Royo, et al., 2007](#_ENREF_3)) |
| MPO95 | 14028 ∆*trg:: nahR/*P*_sal_–xylS2*/P*_Tac_–gfp* fusion | ([Mesa-Pereira, et al., 2013](#_ENREF_2)) |
| MPO375 | SL7207 ∆*trg:: nahR/*P*_sal_–xylS2* /P*_Tac_–gfp* fusion | This work |
| MPO376 | MPO375 ∆*purD::kan* | This work |
| MPO377 | MPO376 ∆*purD* | This work |
| MPO378 | MPO377 ∆*purD*∆*upp::cat* | This work |
| **Plasmid** |  |  |
| pMPO16 | Ap^R^, pCAS derivate with a Pm-*codA* (CD_GUG_) fusion, ColE1 replication origin | ([Royo, et al., 2007](#_ENREF_3)) |
| pMPO20 | Ap^R^, pWSK29 derivative containing a modified MCS, pSC101 replication origin | ([Medina, et al., 2011](#_ENREF_1)) |
| pMPO52 | Ap^R^, pCAS derivative with rrnBT1T2-Pm-T7 SD sequence-MCSII | ([Medina, et al., 2011](#_ENREF_1)) |
| pMPO54 | Ap^R^, pMPO20 derivative with rrnBT1T2-Pm-MCSII | ([Medina, et al., 2011](#_ENREF_1)) |
| pMPO88 | Ap^R^, pMPO52 derivative with rrnBT1T2-Pm- T7SD sequence- *codA* (CD_7AUG_) | This work |
| pMPO90 | Ap^R^, pMPO20 derivative with rrnBT1T2-Pm- T7SD sequence- *codA* (CD_7AUG_) | This work |
| pMPO1088 | Ap^R^, pMPO20 derivative with rrnBT1T2-Pm- *codA* (CD_GUG_) | This work |

**References**

Medina, C., Camacho, E.M., Flores, A., Mesa-Pereira, B., and Santero, E. (2011) Improved expression systems for regulated expression in Salmonella infecting eukaryotic cells, *PLoS One* **6**: e23055.

Mesa-Pereira, B., Medina, C., Camacho, E.M., Flores, A., and Santero, E. (2013) Novel tools to analyze the function of Salmonella effectors show that SvpB ectopic expression induces cell cycle arrest in tumor cells, *PLoS One* **8**: e78458.

Royo, J.L., Becker, P.D., Camacho, E.M., Cebolla, A., Link, C., Santero, E., and Guzman, C.A. (2007) In vivo gene regulation in Salmonella spp. by a salicylate-dependent control circuit, *Nat Methods* **4**: 937-942.
